# Supplementary material for: Are giant clams (Tridacna maxima) distractible? A multi-modal study
Source: PeerJ. 2020 Oct 5;8:e10050. doi: 10.7717/peerj.10050 (PMC7543721; doi:10.7717/peerj.10050)
Supplement: Table S1 — Cohen’s d values were calculated for (A) Number of partial retractions, (B) Latency to close, and (C) Latency to reemerge. [file peerj-08-10050-s001.docx]

**Cohen’s d values calculated from the estimated marginal means.**

Cohen’s d values were calculated for (a) Number of partial retractions, (b) Latency to close, and (c) Latency to reemerge.

a) Number of partial retractions

| Comparison | Cohen’s d |
| --- | --- |
| Control-Flow | -1.471 |
| Control-Sound | -0.914 |
| Control- Flow and Sound | -1.458 |
| Flow- Sound | 0.556 |
| Flow- Flow and Sound | 0.013 |
| Sound- Sound and Flow | -0.544 |

b) Latency to close

| Comparison | Cohen’s d |
| --- | --- |
| Control-Flow | -0.058 |
| Control-Sound | -0.053 |
| Control- Flow and Sound | -0.346 |
| Flow- Sound | 0.005 |
| Flow- Flow and Sound | -0.288 |
| Sound- Sound and Flow | -0.293 |

c) Latency to reemerge

| Comparison | Cohen’s d |
| --- | --- |
| Control-Flow | 0.476 |
| Control-Sound | -0.054 |
| Control- Flow and Sound | -0.088 |
| Flow- Sound | -0.530 |
| Flow- Flow and Sound | -0.564 |
| Sound- Sound and Flow | -0.034 |
